# Partial Splenectomy and Splenic Wrapping for a High-Grade Splenic Injury: A Case Report

**DOI:** 10.7759/cureus.54372

**Published:** 2024-02-17

**Authors:** Jason Zouki, Damian Fry

**Affiliations:** 1 General Surgery, Toowoomba Hospital, Toowoomba, AUS; 2 General Surgery, Darling Downs Hospital and Health Service, Toowoomba, AUS

**Keywords:** general trauma surgery, trauma laparotomy, splenic trauma, splenorraphy, spleen preservation

## Abstract

The spleen is one of the most commonly injured organs in blunt abdominal trauma, accounting for a vast portion of solid organ injuries, and may lead to rapid haemodynamic instability, requiring urgent operative intervention. Total splenectomies result in relative immunocompromise, with a risk of overwhelming post-splenectomy infection (OPSI) post splenectomy. This case reports the surgical management of a 20-year-old male with a grade IV splenic laceration after a motor vehicle accident. The patient underwent a trauma laparotomy with a partial splenectomy because of early take-off of the upper-lobar branch of his splenic artery, with an absorbable mesh wrap to tamponade the spleen. The patient avoided the need for a total splenectomy and was discharged after six days in the hospital with an uncomplicated recovery.

## Introduction

Splenic trauma accounts for 32% of solid organ injuries with significant consequences including haemodynamic compromise and death [1]. Splenectomy is the gold-standard operation in deteriorating patients with splenic injury; however, it carries the risk of relative post-operative immunocompromise and overwhelming post-splenectomy infection (OPSI), which occurs in around 0.5% of adults within the first two years post splenectomy [2]. Splenorraphy refers to surgical repair of the spleen with successful preservation of splenic function in 40% of trauma cases [3]. With increasing access to management options such as interventional radiology, the rate of splenectomies is decreasing; however, in the correct patient group or settings with no access to interventional radiology, splenorraphy is a viable option to safely preserve splenic function.

## Case presentation

A 20-year-old male was transferred via ambulance to a regional hospital in July 2022, 10 hours post motor vehicle accident at 100 km/hr. The patient reported no significant medical or surgical history. On initial assessment, the patient was hypotensive with a blood pressure of 94/70 mmHg, he had a new oxygen requirement with saturations of 91%, and he was tachypnoeic at 24 breaths per minute. A secondary survey revealed right and left upper quadrant (LUQ) abdominal pain with a bedside eFAST (extended Focused Assessment with Sonography in Trauma) positive for free fluid in the LUQ. An initial venous blood gas noted significant anaemia with a haemoglobin of 94 g/L.

**Figure 1:**
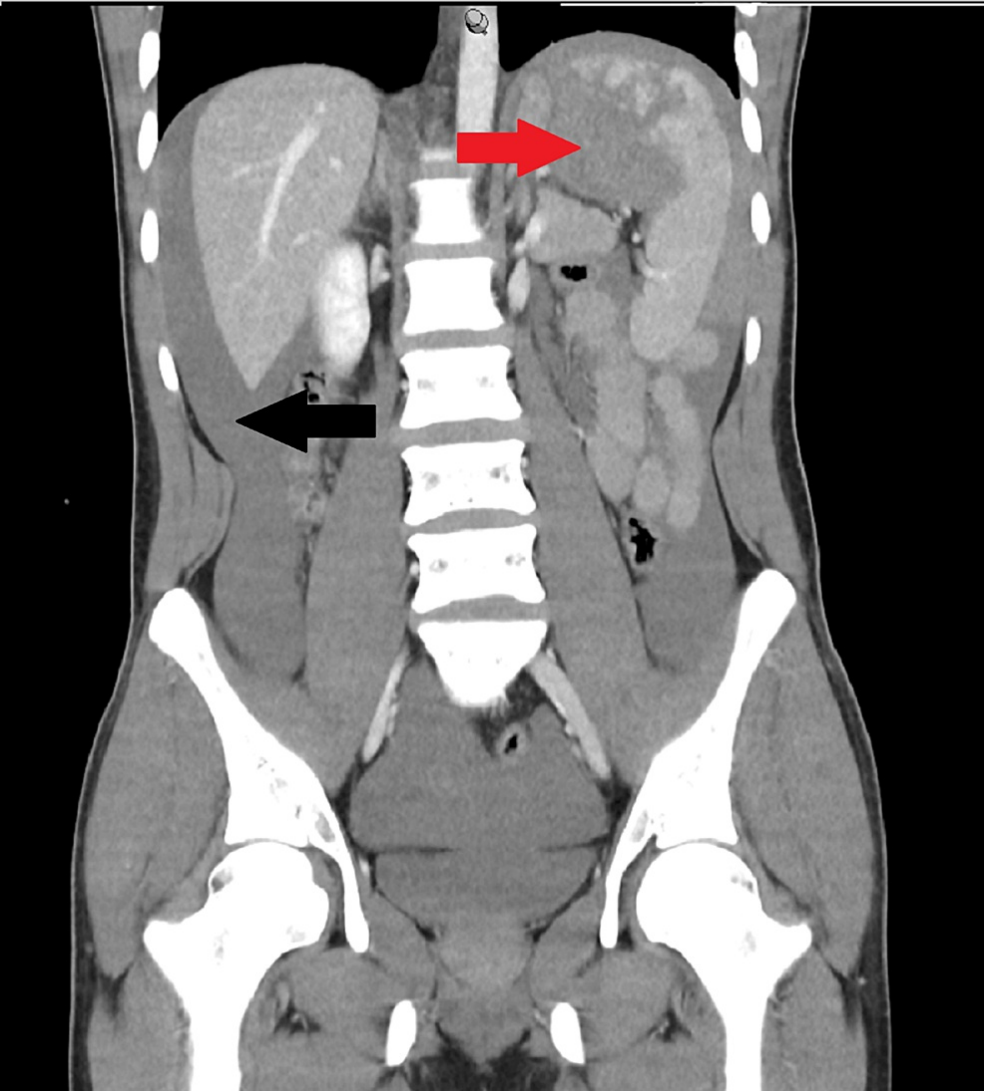
Abdominal CT showing significant haemoperitoneum and Grade IV splenic injury involving the upper pole Red arrow: Grade IV splenic injury producing >25% devascularisation of the spleen Black arrow: Significant haemoperitoneum

A trauma CT scan confirmed a large-volume haemoperitoneum with a 5 cm laceration of the superior splenic pole (Grade IV AAST), as seen in Figure 1, as well as a pulmonary haemorrhage and small bilateral pneumothoraces. The patient was stabilised with a rotational thromboelastometry (ROTEM)-guided blood transfusion and taken to theatre for a trauma laparotomy where the devascularised upper pole of the spleen was resected with a stapler and haemostasis achieved with a topical haemostatic matrix.

**Figure 2:**
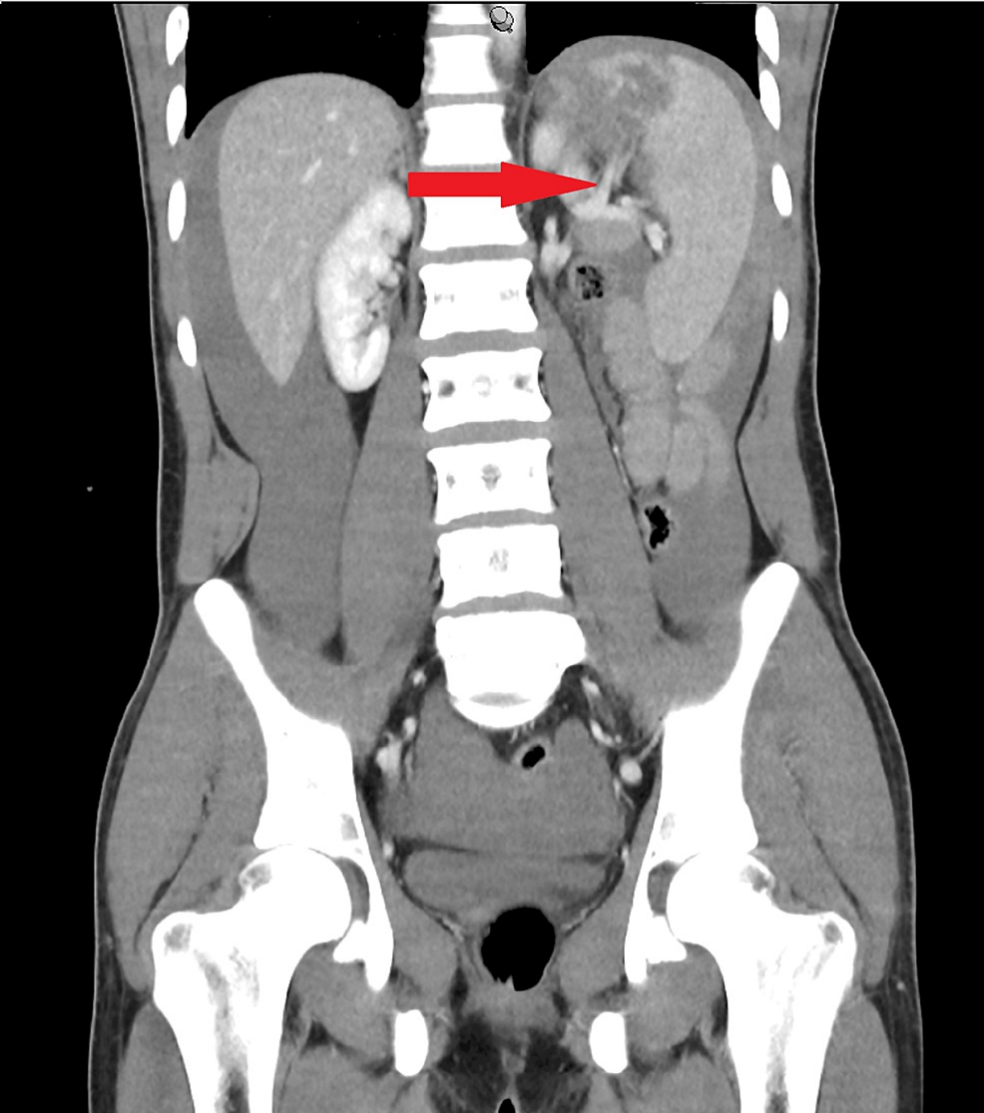
Abdominal CT scan showing splenic injury and involvement of the upper-lobar branch of the splenic artery Red arrow: Upper-lobar branch of the splenic artery extending into upper pole laceration and area of devascularisation

The patient was noted to have a relatively early take-off of the upper-lobar branch of the splenic artery, which was involved in the lacerated upper pole injury, as seen in Figure 2. As such, this branch was staple ligated to ensure adequate resection of the lacerated segment and complete compression of the spleen. The spleen was then completely wrapped in a Vicryl (polyglactin 910; Ethicon, Inc., Raritan, NJ) mesh with the edges collected medially and stapled together as a tamponade. The patient was discharged home with no complications after six days in the hospital.

## Discussion

Splenic injuries are common in blunt abdominal trauma with a severity grading based on the AAST [4]. Management of splenic injuries is divided into operative and non-operative approaches, with consideration given to haemodynamic stability, transfusion requirements, AAST grade, the presence of other abdominal injuries, access to interventional procedures, and patient frailty [5,6]. Furthermore, an asplenic state is associated with relative immunocompromise and a lifetime risk of OPSI of around 1%, with a mortality of around 50% [2]. Non-operative management may preserve splenic function and reduce ongoing infection risks to patients. The role of angioembolisation has increased the success of non-operative management by above 90%; however, associated complications can include a splenic infarct, splenic abscess, and persistent haemorrhage requiring ongoing operative intervention [7].

Splenorraphy as an alternative in trauma has progressed to include splenic wrapping with successful reports of pre-made mesh bags or Dexon (polyglycolic acid) mesh wrapping [8-10]. Patient selection is a key determinant of the type of operative intervention, and the ability of patients to tolerate a complication should be considered when opting for a splenorraphy over a splenectomy. This case presents a non-comorbid patient, with a high-grade splenic injury who had ongoing haemodynamic compromise despite adequate resuscitation. Furthermore, there was no access to interventional procedures at this centre, and a decision was made to attempt a splenorraphy. Key factors that contributed to the operative decisions in this case included the patient’s age and lack of comorbidities, which served as an indicator of his ability to tolerate further operations including a salvage splenectomy in the context of rebleeding if required. The decision for partial resection was because of the shatter injury to the spleen and the inability to gain appropriate compression given the early division of the upper lobar splenic artery branch. The spleen was then wrapped in an absorbable mesh with circumferential compression by joining all edges of the mesh towards the splenic hilum. Tension was maintained by stapling the edges together, whilst allowing a small window at the hilum for the splenic vessels to enter and exit. Haemostasis was successfully achieved by the end of the case, and the patient resolved without complication.

## Conclusions

Splenic injuries are common in blunt trauma and account for a significant portion of splenectomies. In select cases involving robust patients or in hospitals with no access to interventional procedures, a partial splenectomy is an appropriate alternative that can preserve splenic function. Splenic wrapping with an absorbable mesh is an effective way to achieve adequate haemostasis and should be used by trauma surgeons as means of splenic preserving surgery in suitable patients.
